# Exploring New Drug Targets for Type 2 Diabetes: Success, Challenges and Opportunities

**DOI:** 10.3390/biomedicines10020331

**Published:** 2022-01-31

**Authors:** Abhinav Kanwal, Navjot Kanwar, Sanjay Bharati, Prateek Srivastava, Shailendra P. Singh, Salomon Amar

**Affiliations:** 1 Department of Pharmacology, All India Institute of Medical Sciences (AIIMS), Bathinda 151001, India; abhinavkanwal@gmail.com; 2 University Institute of Pharmaceutical Sciences, UGC Centre for Advanced Studies, Panjab University, Chandigarh 160014, India; kanwar_navjot@yahoo.com; 3 Department of Nuclear Medicine, Manipal College of Health Professions (MCHP), Manipal Academy of Higher Education (MAHE) Manipal, Manipal 576104, India; sanjay.bharati@manipal.edu; 4 Chitkara University Institute of Engineering and Technology, Chitkara University, Rajpura 140401, India; prateeksrivastava028@gmail.com; 5 Department of Pharmacology, New York Medical College, Valhalla, NY 10595, USA; 6 Department of Biomedical Engineering, Central University of Rajasthan, Ajmer 305817, India

**Keywords:** type 2 diabetes, diabetes mellitus, drug development, drug discovery, lead molecules, new targets

## Abstract

There are substantial shortcomings in the drugs currently available for treatment of type 2 diabetes mellitus. The global diabetic crisis has not abated despite the introduction of new types of drugs and targets. Persistent unaddressed patient needs remain a significant factor in the quest for new leads in routine studies. Drug discovery methods in this area have followed developments in the market, contributing to a recent rise in the number of molecules. Nevertheless, troubling developments and fresh challenges are still evident. Recently, metformin, the most widely used first-line drug for diabetes, was found to contain a carcinogenic contaminant known as N-nitroso dimethylamine (NDMA). Therefore, purity and toxicity are also a big challenge for drug discovery and development. Moreover, newer drug classes against SGLT-2 illustrate both progress and difficulties. The same was true previously in the case of glucagon-like peptide-1 receptor agonists and dipeptidyl peptidase-4 inhibitors. Furthermore, researchers must study the importance of mechanistic characteristics of novel compounds, as well as exposure-related hazardous aspects of current and newly identified protein targets, in order to identify new pharmacological molecules with improved selectivity and specificity.

## 1. Epidemiology

Diabetes mellitus (DM) is a chronic progressive metabolic disorder in which the body is unable to utilize glucose [1]. It could be caused by a decrease in pancreatic cell insulin secretion or a lack of insulin responsiveness in the body. Insulin aids glucose assimilation within cells, allowing blood glucose levels to remain within a healthy range (80–120 mg/dL). Thus, a deficit of insulin in the body results in hyperglycemia, raising the blood glucose level, which in turn leads to many metabolic and life-threatening complications, including cardiovascular, nephropathic, and neuropathic diseases, amongst others [1].

A significant portion of the world’s population is affected by diabetes mellitus (DM). Studies estimate that 8.8% of those between the ages of 20–79 in the world’s population are diabetic [2]. According to an International Diabetes Federation (IDF) report [2], the Federated States of Micronesia (Micronesia) have the world’s most overweight population, with a 30% obesity prevalence [3]. On the other hand, North American and Caribbean adults aged between 20–79 years have the highest prevalence (13%) of diabetes among Western IDF regions [2]. In South Asian countries, diabetes prevalence is at its highest in Mauritius (22%), followed by Sri Lanka (10.7%), and India (10.4%) [2]. Approximately 425 million people worldwide are affected by diabetes, and this is projected to increase to ~629 million by the year 2045 due to unhealthy diets and inactive lifestyles [4].

In the case of Micronesia and other nearby islands, there is one hypothesis that argues the cause of severe diabetes is inadequate physical activity and an unhealthy diet. During times in which there is an inadequate food supply, people in Micronesia consume an excess amount of sugar to sustain themselves. After recovering from such periods, even with an increased availability of higher quality foods, sugar consumption has become an integral part of their daily dietary habits. Micronesian survey data show that around 73.1% of the population is overweight, and 32.1% frankly diabetic [5].

DM is a major threat to both the developed and developing worlds. According to the 2017 National Diabetes Statistics Report, about 30.3 million people in the U.S. are diabetic and surprisingly some 23.8% are undiagnosed [2]. The latest 2019 statistics by the IDF are shown in Table 1. However, in countries like India, the dominant factor contributing to the prevalence of diabetes is an underdeveloped health services system, as 70% of the Indian population lives in poorly serviced rural areas. Widespread illiteracy and unfamiliarity with healthier nutritional habits only serve to exacerbate the situation. These factors contribute to inadequate diabetes screening and availability of preventive measures, as well as non-adherence to diabetes management guidelines once diagnosed [6]. Less physical activity and sedentary lifestyles may also contribute to obesity and T2DM. Correspondingly, increased physical activity has been shown to help maintain glucose homeostasis in the body and to delay the onset of impaired glucose tolerance [7].

## 2. Classification of Diabetes Mellitus

Diabetes existed long before its mechanisms were understood. The first reference to diabetes was in 1550 B.C. in an Egyptian papyrus, where it was described as a rare disease characterized by excessive weight loss and frequent urination [8]. The term diabetes (to siphon or to pass through, in Greek) was first used by Apollonius of Memphis, who noted that patients with diabetes present with frequent urination [9]. A Greek physician, Galen, hypothesized it to be a type of kidney disease, as the most prominent symptom was glucosuria. Avicenna (980–1037) offered a comprehensive description of diabetes mellitus in his publication “The Canon of Medicine” in 1025 [10]. Eventually, owing to its characteristic symptoms, many tests to more fully identify the disease were developed in the late 11th century, involving the examination of the subject’s urine’s color, odor and taste. The sweet taste of a diabetic patient’s urine apparently led to its second name, mellitus (honey, in Latin) [9]. Later in the 19th century, Matthew Dobson discovered that the excessive amount of sugar in the urine of patients with diabetes gives it that sweet taste. He also provided an important distinction between the two different types of diabetes, which are now referred to as type 1 and type 2 diabetes. Banting and Macleod observed a reduction in blood glucose levels after administering islets of Langerhans to a pancreatectomized dog, and for this breakthrough discovery they were awarded the Nobel Prize in 1923 [11]. Eli Lilly acquired the first deal to produce insulin on a commercial level. Gradually, by the late 20th century, as the mechanisms behind diabetes started to unfold, various insulin preparations and other oral hypoglycemic drugs paved their way onto the market.

At present, based on insulin deficiency and insensitivity of cells towards insulin, diabetes is classified into three major types, described below. The detailed classification can be found in the WHO’s classification of diabetes mellitus 2019 [12].

### 2.1. Type 1 Diabetes Mellitus

T1DM is primarily caused by the destruction of pancreatic β-cells by the body’s immune system and is also known as Insulin Dependent Diabetes Mellitus. This type of diabetes is found across all age groups of patients, but the prevalence is higher among children [13].

### 2.2. Type 2 Diabetes Mellitus

T2DM is the most common type of diabetes. It is a chronic and multifactorial disease. Earlier this was also called Non-insulin Dependent Diabetes Mellitus (NIDDM), but this term is no longer used because insulin is often used in the management of NIDDM. T2DM is believed to be caused by the gradual development of cells resistant towards insulin and β-cell dysfunction, although the exact mechanism remains unclear. Being overweight or obese is a major risk factor for T2DM, as it may increase the likelihood of insulin resistance. This in turn will decrease uptake of glucose in, for example, the heart or musculoskeletal tissues, with simultaneous increase in glucose production in organs such as the liver [14]. To counter this, insulin secretion is enhanced by β-cells. Therefore, hyperglycemia and hyperinsulinemia often coexist during early T2DM [15]. In later stages of the disease, β-cell function is also diminished. Thus, therapeutics that target β-cell functioning and decrease insulin resistance are key treatments in T2DM. Some of the factors that control β-cell functioning include oxidative stress, ER-stress and autophagy. Over the years, researchers have worked on various targets to moderate the onset or worsening of diabetes in their patients, out of which many turned into drug targets.

### 2.3. Gestational Diabetes Mellitus (GDM)

Mothers with GDM is a type of glucose intolerance that occurs during pregnancy. It affects around 2–14% of pregnant women worldwide [16]. Mothers with GDM are at increased risk for the development of T2DM after pregnancy. Their offspring have increased rates of birth trauma, as well as a higher chance of developing issues with obesity and diabetes later in life. Mothers with GDM can be managed by diet and exercise with only some cases requiring medication.

## 3. Common Targets for Anti-Diabetic Drug

T1DM is managed by regular administration of insulin and modifications in the diet and lifestyle of affected individuals [17]. T2DM is the most common type of diabetes, and due to its complex pathogenesis, the ideal treatment for T2DM has been difficult to find. Several studies have reported compounds that improved insulin’s action on target tissues and helped restore β-cell function. In the last decade, many targets have been discovered as new oral agents for T2DM patients [18]. Among these, four major target types are known as insulin secretagogues, insulin mimickers and sensitizers, and starch blockers.

### 3.1. Insulin Secretagogues

Insulin secretagogues work by stimulating β-cells to secrete more insulin. There are two types: sulfonylureas and non-sulfonylureas. Sulphonylureas (SU) bind to the sulphonylurea receptors on pancreatic β-cells and stimulate them to secrete insulin. SU have shown good efficacy in reducing glycated hemoglobin microvasculature complications in patients with T2DM. However, their major drawback is prolonged binding time to the β-cells, which results in prolonged insulin release. The non-sulfonylureas also stimulate β-cells to secrete insulin, but they are short acting. In 2004, Jean-Claude Henquin proposed five potential action sites for insulin secretagogue β-cell metabolism activation: increased blocking of β-cell K_ATP_ channels; increase in intracellular (Ca^2+^) concentration by means other than K_ATP_ channels; activation of various amplifying pathways in β-cells; action on β-cell membrane receptors; and action on β-cell nuclear receptors [19].

### 3.2. Insulin Mimickers and Sensitizers

Insulin mimickers and sensitizers are agents which are helpful in lowering blood-glucose levels and are usually found as dietary supplements. They work by activating glucose transporters on muscle and fat cells, thus mimicking the function of insulin. Insulin sensitizers work by increasing the sensitivity of body tissues towards insulin. A variety of observable cardiac risk factors such as an increased risk of blood clotting, elevated blood pressure, lipid profile or C-reactive protein, undesired lipoprotein(a) or serum fibrinogen, and even abnormal thickening of the heart muscle have also been shown to improve upon use of insulin sensitizers [20].

### 3.3. Starch Blockers

Another important class is alpha-glucosidase inhibitors, which act by slowing down carbohydrate absorption. The most common drugs are Acarbose and miglitol [21]. Another category is dipeptidyl peptidase-4 (DPP-4) inhibitors, which also act in carbohydrate metabolism. For example, Glucagon-like peptide-1 (GLP-1) is released after meal ingestion and found highly expressed in adipose tissue. It works by delaying gastric emptying, increasing insulin secretion, and reducing glucagon secretion [22].

**Figure 1:**
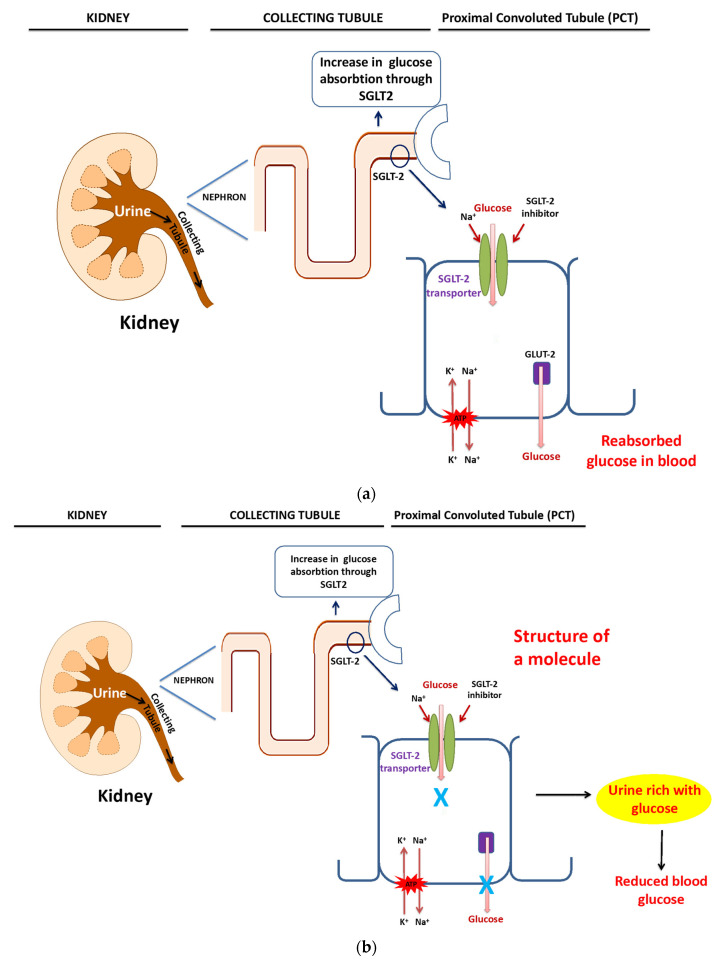
(**a**) Sodium-glucose co-transporter type 2 (SGLT2) inhibitor pathway. (**b**) Sodium-glucose co-transporter type 2 (SGLT2) inhibitor pathway.

Sodium-glucose co-transporter type 2 (SGLT2) inhibitors are another recently discovered class. Canagliflozin and dapagliflozin are recently approved drugs in this category. These agents promote glucose excretion by the kidney and thus exert their actions independent of insulin (Figure 1a,b).

The other advantages of these drugs include modest weight loss and a low risk of hyperglycemia, etc. [23]. The search for the ideal anti-diabetic drug is still ongoing, leading to the development of newer molecules which are in different phases of clinical trials. Some of the anti-diabetic drugs at phase-III clinical trials are listed in Table 2.

## 4. Emerging Targets for the Treatment of T2DM

### 4.1. 11β-Hydroxysteroid Dehydrogenase

11β-Hydroxysteroid dehydrogenase is an enzyme that catalyzes the conversion of inactive cortisone to active cortisol. Literature reports indicated that high circulating levels of active glucocorticoid cortisol contribute to various disorders such as diabetes mellitus, obesity, dyslipidemia and high blood pressure. The 11β-HSD knockout transgenic mice were shown to have higher insulin sensitivity and were comparatively more resistant to high fat induced obesity, whereas studies also suggested that the over expression of 11β-HSD made animals prone to metabolic syndrome [24]. Hence, 11β-HSD is considered an important drug target for T2DM.

### 4.2. Glutamine Fructose-6-Phosphate Amido Transferase

As reported earlier, most glucose entering the cell undergo glycolytic metabolism and a very small percentage enter the hexosamine pathway. Biosynthesis of hexosamine is well known for its contribution to insulin resistance and activation of growth factor synthesis. Enzyme Glutamine fructose-6-phosphate aminotransferase (GFAT) has a central role in the hexosamine biosynthetic pathway, as it is involved in catalysis of the first and rate limiting steps for hexosamine formation. Therefore, it is considered to be an important therapeutic target against T2DM [25].

### 4.3. Protein Tyrosine Phosphatase 1B

Protein tyrosine phosphatases, such as protein tyrosine phosphatase 1B (PTP1B) and leukocyte antigen-related tyrosine phosphatase, play an important role in modulating insulin signal transductions [26]. Tyrosine phosphorylation in the insulin-receptor activation loop is an important step in insulin signal transduction. PTP1B dephosphorylates phosphor-tyrosine residues of insulin receptor kinase activation segments leading to negative regulation of insulin signaling. The evidence further suggests a relationship between PTP1B, insulin sensitivity, obesity and T2DM. PTP1B is also a negative regulator for leptin signaling [1]. Leptin is a hormone primarily involved in maintaining metabolic homeostasis, and in obesity a decreased sensitivity to leptin results in the inability to attain satiety despite having good energy stores. PTP1B also plays an important part in pancreatic β-cell proliferation. For example, Fernandez-Ruiz et al. found that that PTP1B knockout mice have a higher proliferation of β-cells and elevated glucose-induced insulin secretion [27]. These studies are strongly suggestive of the role of PTP1B in diabetes, and thus interest for PTP1B inhibitors, resulting in the search for and development of several inhibitors of this protein. Detailed descriptions of the reported PTP1B inhibitors can be found elsewhere [28].

### 4.4. SLC16A11

The genome-based studies carried out by the Slim Initiative in Genomic Medicine for the Americas (SIGMA) Type 2 Diabetes identified a genetic locus which is strongly associated with T2DM. These diabetes-associated haplotypes caused a decrease in SLC16A11 expression in the liver and disrupted interaction with basigin, which reduced the cell surface localization of SLC16A11 [29]. SLC16A11 knockdown in primary human hepatocytes leads to modulation of fatty acid and lipid metabolism [29]. This modulation increases intracellular acylcarnitine, diacylglycerol and triacylglycerol levels which results in increased triglycerides in blood circulation and an accumulation in liver tissue [30,31]. The increased levels of triglycerides are implicated in insulin resistance, so variants of SLC16A11 may increase the risk of diabetes by regulating lipid metabolism. However, many questions remain unanswered, for example, what the unique substrates are that are associated with the transport of mediators targeting the physiological and biochemical mechanisms that affect T2DM.

### 4.5. Nephroblastoma Overexpressed (CCN3/NOV)

CCN3 (also nephroblastoma overexpressed) is a cysteine-rich protein which is involved in growth-regulatory functions. Its presence is noted in a variety of human tissues and biological fluids such as the musculoskeletal system, kidneys, and cerebrospinal fluid [32]. The average plasma levels of CCN3 have a strong correlation with hs-CRP, BMI, and fat mass, and are elevated significantly in obese patients with hyperlipidemia [33]. Martinerie et al. demonstrated that CCN3 deficiency in mice fed with standard high-fat diets markedly lowered their body weight, and improved glucose tolerance and insulin sensitivity [34]. Moreover, Li et al. investigated the serum CCN3 levels in newly diagnosed T2DM (nT2DM) patients and compared it with healthy control subjects [35]. It was observed that CCN3 levels were significantly elevated in T2DM patients. These data suggest CCN3 may be involved in obesity-associated insulin-resistance and can serve as an important target for a T2DM therapeutic [35].

### 4.6. FoxO1

The forehead transcription factor FoxO1 is another important target of T2DM and a prominent mediator of insulin signaling in β-cells. It has been shown that dominant-negative FoxO1 adipocytes improved both glucose as well as insulin tolerance in a high-fat diet model. In addition, Fox01 in the pancreas are responsible for β-cell dysfunction by inducing stress and apoptosis. Phosphorylation and acetylation are the two most common post translational modifications of FoxO1, where it regulates many gene functions [36]. Phosphorylation of FoxO1 is the main mechanism by which it comes out of the nucleus to get degraded by ubiquitination. The acetylation state of FoxO1 depends upon the balance between the protein acetylases and deacetylases. Even FoxO1 gets activated by O-GlcNAcylation during oxidative stress-like conditions in diabetic livers and increases the activation of many gluconeogenic and ROS detoxifying genes [36]. PGC1-α induction during fasting activates the series of gluconeogenesis genes and later directly binds to FoxO1 to phosphorylate and ultimately degrade it out of the nucleus.

### 4.7. FFA2/FFA3

The free fatty acids (FFAs) can act as signaling molecules. FFAs are generally divided into three sub-categories based on their chain length as short-chain fatty acids (SCFAs), medium-chain fatty acids (MCFAs) and long-chain fatty acids (LCFAs). FFAs of these varying chain lengths are known to activate transmembrane receptors such as FFA1, FFA2, and FFA3. For example, FFA1 are expressed to a large extent on the pancreatic β-cells and are activated by LCFAs. The activation of these receptors also plays an important role in the increase of glucose-stimulated insulin secretion. Therefore, because of its obvious role in glucose-stimulated insulin secretion, a series of FFA1 ligands have been identified and tested. FFA2 and FFA3 are akin to FFA1 receptors and get activated by SCFAs but their role in insulin is complex. FFA2 receptors are highly expressed in immune cells, especially neutrophils [37,38,39]. Studies have also identified FFA2 expression on murine pancreatic β-cell line MIN6 and isolated mouse islets [40,41]. The indirect support for the role of FFA2 in diabetes comes from the fact that a fiber-rich diet is linked with a lower incidence of diabetes and SCFAs. Ligands for FFA2 are known to be produced by intestinal bacteria in the body by the fermentation of dietary fibers [42]. Tang et al. [43] showed FFA2 and FFA3 expression on human pancreatic β-cells. These receptors are shown to inhibit insulin secretion by coupling to Gi-type G proteins. They also demonstrated that genetic deletion of FFA2 and FFA3 receptors in pancreatic β-cells leads to higher insulin secretion in HFD animals, whereas similar deletion in intestinal cells did not affect glucose tolerance in diabetic animals. This study demonstrated that FFA2/FFA3 antagonists may prove beneficial in T2DM [43].

### 4.8. Epoxyeicosatrienoic Acids (EETs)

Epoxyeicosatrienoic acids (EETs) are produced from arachidonic acid by cytochrome p450 enzymes (monoxygenase/epoxygenase) in vascular endothelium responses to various stimuli, such as the agonists acetylcholine (ACH) or bradykinin, or by shear stress which activates phospholipase A2 to release arachidonic acid [9]. The cytochrome p450 enzyme is responsible for the formation of 20-hydroxyeicosatetraenoic acid (20-HETE) and EETs. The soluble epoxide hydrolases and reactive oxygen species (ROS) induction rapidly hydrolyze the EETs into their respective dihydroxyepoxytrienoic acids (DHETs) as well as esterificate primarily to glycerophospholipids [44]. The literature shows that EETs demonstrate anti-inflammatory, vasodilatory, and anti-apoptotic actions, inhibited sEH significantly, and elevated EETs’ cellular and circulation levels [45,46,47].

The administration of EET or inhibitors of sEH to obese mice is associated with a decrease in visceral subcutaneous fat and an increase in insulin sensitivity. The CYP2J2-mediated increase in EET increases fatty acid oxidation and adiposity. Although EETs have a naturally robust action, including vasodilatation and restraint of the inflammatory response, in adiposity, their effect on mitochondrial work and proliferator-actuated receptor gamma coactivator-1α (PGC-1α) corresponding to adipogenesis is still unclear [46,48,49,50].

### 4.9. Peroxisome Proliferator-Activated Receptor Gamma Co-Activator Alpha (PGC-1α)

PGC-1α is a protein encoded by the PPARGC1A gene in humans. PGC-1α maintains energy homeostasis and regulates the expression of insulin signaling, mitochondrial biogenesis, dynamics, and antioxidant genes, including uncoupling proteins, and thus prevents mitochondrial dysfunction and metabolic disorders related to adipocyte malfunction [51]. Dysregulation of PGC-1α alters homeostasis in cells and exacerbates the inflammatory response, which is commonly accompanied by metabolic disturbances. During adipocyte dysfunction, low degrees of PGC-1α downregulate mitochondrial quality articulation, instigate irritation and oxidative pressure, and advance atomic factor κ-B actuation [52].

PGC-1α quality treatment facilitated improved fat tissue work and had a positive and beneficial effect on distal organs like the liver, suggesting that focusing on PGC-1α quality treatment is an alluring remedial methodology for improving insulin affectability, insulin processing, metabolic movement and vascular capacity in metabolic disorders [53].

### 4.10. Peroxisome Proliferator-Activated Receptor Gama (PPAR©)

One of the most important nuclear targets for TZDs is PPAR©. Originally, it was considered the positive regulator of adipogenesis with higher expression in adipose tissues, though it is also expressed in many other tissues and body cells of the body but to a lesser extent. It has been observed that specific deletion of PPAR© from different cell targets—such as adipose tissue, muscle, macrophages, and the brain—alters glucose homeostasis, which in turn reduces the activity of TZDs. PPAR© is well known for both promoting and secreting adiponectin which is considered an important insulin sensitizing hormone [36]. It is a well-known transcription factor that regulates the functions of many genes both positively and negatively. However, fully understanding PPAR©’s role and how it controls insulin sensitivity is still elusive. Despite many side effects, which include heart failure, weight gain, bladder cancer, plasma volume expansion, and others, it is still considered an important target. Understanding its mechanism and exploring its downstream pathway is of great interest. Epigenetic modifications or post translational modification in PPAR©’s structure are thought to be promising routes in the treatment of various metabolic diseases. Various studies, including those on its phosphorylation at S273 or sumoylation at Lys107, have already demonstrated its importance in mediating insulin sensitizing actions [36]. Sirtuins-mediated changes in PPAR© also were beneficial in metabolic syndrome.

### 4.11. Glucocorticoid Receptor

The glucocorticoid receptor has a distinct effect on insulin action with both endogenous as well exogenous synthetic glucocorticoids [54]. In general, glucocorticoids increase the blood glucose levels when required by the brain, but chronic activation of these receptors is associated with metabolic dysfunction, including insulin resistance [36]. Glucocorticoids exhibit their effects either by directly acting on promoter or enhancer sites of the genes or by acting on other transcription factors and making a complex. Homodimerization and direct binding are associated with insulin resistance, affecting many gluconeogenic genes such as Pck1 and G6pc [36]. Chronic activation of these receptors inhibits protein synthesis and favors proteolysis. This ultimately releases amino acids and acts as substrate for glucose production. While the precise mechanism for how these receptors regulate insulin resistance is still unknown, many studies show that GR activation inhibits insulin stimulated glucose uptake by controlling many downstream targets.

### 4.12. Nuclear Factor (Erythroid-Derived 2)-Like 2 (NRF2)

NRF2 is an important molecular node which imparts cytoprotection in many diseases. Due to its different roles in various diseases, it is predicted to be an important target in drug discovery. With concern to type 2 patients with diabetes, a significant difference was found in the genotypic and allelic frequencies of four SNPs of the NFE2L2 gene. In addition, one study involved induction of NRF2 in obese diabetic db/db mice, displaying a decreased blood glucose level by suppressing hepatic glucose 6 phosphatase via cAMP-CREB signaling [55]. Leptin deficient mice showed decreased expression of NRF2 and displayed reduced amounts of white fat mass. This suggests that NRF2 is a key player in adipogenesis or in other metabolic disorders. It has been postulated that NRF2 levels can be increased with an acute dose of glucose, but chronic glucose conditions failed to activate it. Expression is also downregulated in prediabetes patients [55]. NRF2-mediated protection is well defined in the dysfunction of diabetic retinopathy as well. NRF2 knockdown enhances the palmitate-induced apoptosis in hepatocytes of obese patients.

### 4.13. Neprilysin

Neprilysin is a zinc-dependent metalloprotease that cleaves peptides (GLP-1 [7,8,9,10,11,12,13,14,15,16,17,18,19,20,21,22,23,24,25,26,27,28,29,30,31,32,33,34,35,36] amide, GLP-1 [9,10,11,12,13,14,15,16,17,18,19,20,21,22,23,24,25,26,27,28,29,30,31,32,33,34,35,36] amide from GLP-1 [28,29,30,31,32,33,34,35,36] amide and GLP-1 [32,33,34,35,36] amide) and inactivates several peptide hormones including glucagon, enkephalins, substance P, neurotensin, oxytocin, and bradykinin [56]. It was thought that GLP-1 [9,10,11,12,13,14,15,16,17,18,19,20,21,22,23,24,25,26,27,28,29,30,31,32,33,34,35,36] amide, which is the major circulation form in plasma, is the c-terminal metabolite which originates after the enzymatic cleavage of incretin hormone GLP-1 [7,8,9,10,11,12,13,14,15,16,17,18,19,20,21,22,23,24,25,26,27,28,29,30,31,32,33,34,35,36] amide by dipeptidyl peptidase-4. These new metabolites also were considered to have an insulinomimetic activity that contributes to the pleiotropic effects of GLP1, other than its canonical GLP-1R. It has been found that direct administration of these metabolites has antioxidant, anti-apoptotic, and proliferative effects on pancreatic β-cells by modulating energy homeostasis. It has been reported that these metabolites preserve mitochondrial function, inhibit membrane depolarization and caspase activation and hence, apoptosis of INS-1 β-cells. The effect of these metabolites on diabetes is further demonstrated by the administration of GLP-1 [28,29,30,31,32,33,34,35,36] amide, which increases β-cell mass and proliferation by activation of the cAMP/PKA signaling cascade and phosphorylation of the Wnt/β-catenin signaling pathway.

## 5. Drugs Targeting Molecular Pathways Implicated in Diabetes and CVD

In finding new therapeutics the multifactorial nature of T2DM should be considered, as well as prioritizing delivery of treatment at the earliest possible time. In addition, elevated risk of CVD is usually associated with T2DM. Consequently, current FDA guidelines necessitate that all new diabetic therapeutics should demonstrate protective or at least neutral cardiovascular impact profiles. In this way, targets of sub-atomic pathways conceivably involved in both diabetes and CVD are particularly attractive. Such methodologies incorporate the focusing of 11-hydroxysteroid dehydrogenase type 1 (11β-HSD1), GPR119, TGR5, sirtuin 1 (SIRT1), the sodium-glucose co-carrier 2 (SGLT2), and GPR40, for every one of which the reasoning is briefly depicted below (Table 3).

Taken together, these clinical findings give convincing evidence that various inhibitors, activators, agonists, and antagonists hold promise as new treatments for T2DM and related metabolic disorders.

## 6. Diabetes and the Gut Microbiota: Dietary Influence

Diet is a key factor influencing the composition of the gut microbiome, indicating the potential for therapeutic dietary strategies to manipulate microbial diversity, composition, and stability. Although changes in gut microbiota are temporary, diet can induce a shift in the gut microbiota. Numerous studies have pointed to a direct correlation between short-term dietary changes as well as long-term dietary changes with gut microbiome diversity in mouse models. In addition, it was found that gut microbiota participates in drug metabolism by producing specific enzymes, such as reductase and hydrolytic enzymes, thus affecting the efficacy, toxicity, and bioavailability of drugs. Various antibiotics also alter the diversity and composition of gut microbiota which leads to dysbiosis, and it was found to be linked with progression of various neurodegenerative as well as metabolic disorders [73,74,75].

Gut microbiota dysbiosis has been found as a major culprit in initiation as well as progression of various metabolic syndromes including diabetes mellitus, which unlocks various therapeutic interventions for the prevention as well as treatment of metabolic syndrome [76,77].

Isoflavone (genistein) reduced fasting glucose and insulin, as well as HOMA-IR, fibrinogen, and homocysteine levels [78]. However, the current study’s findings must be seen in the context of a healthy, well-balanced diet, which is likely to have boosted the positive benefits of genistein aglycone in our postmenopausal women [79]. Isoflavones have been shown to have anti-diabetic benefits due to their a-glucosidase inhibitory activity. It is thought that genistein prevents diabetes by preserving insulin-positive β-cells and altering the hepatic glucose metabolic enzyme profile. Genistein may prevent glucose-induced lipid peroxidation, modify cell islet pancreatic function, and increase basal metabolic rate and energy metabolism [80]. In vitro and in vivo studies have shown that genistein can serve as an estrogen agonist, resulting in the growth of estrogen-dependent human breast cancer tumors, and that long-term treatment with soy phytoestrogens has been linked to an increased risk of endometrial hyperplasia [81].

## 7. Crosstalk between Diabetes and Fatty Liver Disease-NASH and Prospective Therapeutic Drug Targets

Altered glucose and lipid metabolism are key factors associated with the development of NASH. Studies have shown the close relationship between metabolic disease and NASH [82,83]. People having NASH are more insulin resistant than healthy people, irrespective of their weight. Although most of the patients with NASH are insulin resistant, they do not show hyperglycemia because of decreased ability of the liver to clear insulin. NASH patients only develop diabetes when pancreatic beta cells are unable to increase insulin secretion to match the insulin resistance [84,85,86].

Some of the diabetic medications are used for the management and treatment of NASH. Glucagon-like-peptide-1 (GLP-1) receptors, which are used as second-line therapy for diabetes, have been used for amelioration of insulin resistance in NASH patients which ultimately improves the hepatic condition. The possible mechanism behind the improvement in insulin resistance is changes in body weight. However, researchers are still exploring the exact mechanism of these drugs [87,88]. Dipeptidyl peptidase (DPP)-4 inhibitors are another diabetic lowering class of agents that can be used for the treatment of NASH. Although these agents are not potent glucose-lowering agents due to their oral intake and low safety, these are used in combination with metformin in the management of diabetes. However, strong evidence is still missing for the clinical use of these agents for the management of NASH [89]. Pioglitazone, an anti-diabetic drug, has been shown to improve the condition in 60% of NASH patients. SGLT2 can be used for the treatment of NASH as it is helpful in glucose homeostasis. It has been shown to reverse liver steatosis and hepatocytes necrosis. These agents have been shown to decrease the plasma aminotransferases resulting in weight reduction which ultimately improves liver steatosis. One of these agents has been found to improve beta cell functions and insulin metabolism. Insulin has been under research for the treatment of liver steatosis and improvement in NASH [90].

## 8. Challenges and Opportunities

**Figure 2:**
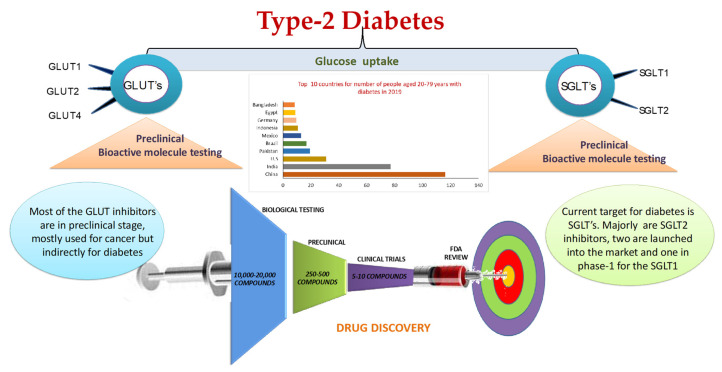
Timeline of t2DM Drug Development.

Despite the helpful impacts of current glucose-lowering drugs, morbidity and mortality remain significant in T2DM patients, highlighting the need for the development of pharmacotherapeutics with a special focus on the many metabolic irregularities and different pathways associated with morbidity. Probably the best test in T2DM treatment is to avoid the drawn-out side effects of any therapeutic intervention, including nonalcoholic greasy liver infection/nonalcoholic Steatohepatitis (NAFLD/NASH) and CVD. Forthcoming investigations in T2DM have demonstrated a relationship between the level of hyperglycemia and the danger of micro- and macro-vascular inconveniences, including deadly CVD events. In any case, the new ACCORD and ADVANCE findings in patients with longstanding T2DM have demonstrated that aggressive glucose control in such patients has no reasonable advantages or may even increase the risk of CVD events. In this manner, other independent risk factors may exist that together contribute to CVD risk in these patients. On the other hand, such discoveries may essentially mirror the restrictions of current diabetic treatments, due to off-target impacts that counter the likely advantages of glucose-lowering agents. The discovery of SGLT2 inhibitors that seemingly have only a few drawbacks needs to be further studied. In phase-III clinical trials, vulvovaginal candidiasis and mycotic infections were the most common side effects seen in females. Osmotic diuresis is possible as glucose excreted in the urine also increases water and solute output, which can further lead to ketoacidosis or acidosis by hyperkaliemia and renal insufficiency. On average, SGLT2 inhibitors are expensive and difficult to continue for long-term use (http://www.endocrinologynetwork.com/sglt2/sglt2-inhibitors-pros-cons-comparisons-and-considerations, 15 September 2021). Currently, the search for new drug molecules that can activate sirtuins is of peak interest, and here resveratrol is considered the best characterized compound. However, it does not activate SIRT1 directly. Many similar molecular structures have been made of SRT1720 and SRT2104, which are active. Still, they need some substrate, and surprisingly they activate some fluorophore tags. They activate SIRT1 directly if endogenous sirtuin is similar to the fluorophore substrate [91] (Figure 2).

## 9. Summary

The past thirty years have seen unrest in diabetes treatment optimization, especially in basic insulin treatment for glycemic control. This welcome activity is geared toward greater understanding of the hidden systems of disease pathology and the advancement of a great number of potential therapeutics. Lamentably, sedentary lifestyles and uneven dietary preferences have contributed to the current obesity pandemic. Along these lines, there is a steadily developing need to improve prediabetes and diabetes treatment approaches. While therapeutic options have unquestionably advanced, the International Diabetes Federation reports that many patients with diabetes remain unable or unwilling to follow current clinical care guidelines.

HbA1c-reducing agents are being evaluated in large clinical trials such as ACCORD, ADVANCE and VADT, in which improved control of HbA1c levels did result in reductions of serious microvascular diabetic complications, although rates of macrovascular infection and mortality remained essentially unchanged. It is consequently now understood that future therapeutic approaches should address clinical endpoints consistent with a multifactorial view of T2DM.

A wide variety of methodologies are being deployed to understand the multifactorial nature of T2DM. The ClinicalTrials.gov site records an excess of 1000 active type 2 diabetes trials around the world. These studies include research focused on cardiovascular involvement and liver diseases, among many others. By and large, the coming years hold great promise for the development of novel therapeutic interventions for T2DM and its related illnesses.

## 10. Future Perspectives

The medications currently available for T2DM have significant limitations and side effects. These notable shortcomings compromise their efficacy across many different clinical endpoints. Therefore, there is an ongoing need to search for new drug targets or combinations of existing drugs.

Future drug and therapeutic developments must follow a holistic approach with special focus on the following points: (1) they should target mechanisms to achieve efficient glycemic control with a safe weight loss profile; (2) they should mitigate the progression of diabetes-induced microvascular and cardiac complications.

Bariatric surgery has provided sensational improvements for many patients; however, significant relapse after six months has been reported. It is important to the scientific community to focus on the factors that may be involved in those early stages of improvement to advance T2DM research. To these ends, various groups have already started enrolling cohorts of obese diabetic and non-diabetic patients in a gastric banding trial. Blood and tissue samples will be collected from these patients (and a complementary control arm) at the time of the surgical procedure and then longitudinally during a five-year follow-up. The clinical and laboratory findings from this and other ongoing trials will no doubt contribute to advancing our therapeutic approaches to the management of diabetes and other metabolic disorders (Table 4).

## Figures and Tables

**Table 1:** World diabetes burden by year 2045.

2019 Rank	Country/Territory	2019 (Millions)	Country/Territory	2045 (Millions)
1	China	116.4	India	134.3
2	India	77	China	119.8
3	U. S. A.	31	U. S. A.	35.6
4	Pakistan	19.4	Mexico	21.8
5	Brazil	16.8	Brazil	20.3
6	Mexico	12.8	Egypt	16.7
7	Indonesia	10.7	Indonesia	16.7
8	Germany	9.5	Pakistan	16.1
9	Egypt	8.9	Bangladesh	13.7
10	Bangladesh	8.4	Turkey	11.2

**Table 2:** New anti-diabetic drugs in the drug discovery pipeline.

Name	Sponsor/Developer	Mechanism of Action	Indication
Afrezza	MannKind	Ultra-rapid-acting mealtime insulin therapy	Adults with type 1 or type 2 diabetes
Albiglutide	GlaxoSmithKline	Glucagon-like peptide (GLP) 1 agonist	Once weekly for adults with type 2 diabetes
Aleglitazar	Roche	Dual peroxisome proliferator-activated receptor (PPAR) α/γ activation	Cardiovascular risk reduction in type 2 diabetes
Alogliptin, Alogliptin and pioglitazone, Alogliptin and metformin	Takeda Pharmaceuticals and Furiex Pharmaceuticals	DPP-4 inhibitor	Oral treatment of type 2 diabetes, individually and in two fixed-dose combinations
Atrasentan	AbbVie	Selective endothelin-A receptor antagonist	Oral once-daily treatment for diabetic nephropathy
Dulaglutide (LY2189265)	Eli Lilly	GLP-1 analog	Once weekly for type 2 diabetes
Empagliflozin (BI10773)	Boehringer Ingelheim and Eli Lilly	Sodium dependent glucose transporter 2 (SGLT2) inhibitor	Oral treatment for adults with type 2 diabetes
Ertugliflozin (MK-8835; PF-04971729)	Merck & Co., licensed from Pfizer	SGLT2 inhibitor	Type 2 diabetes
Fasiglifam (TAK-875)	Takeda	G-protein-coupled receptor (GPCR) 40 agonist	Type 2 diabetes
FIAsp (NN1218)	Novo Nordisk	Faster-acting formulation of insulin aspart	Type 1 and 2 diabetes
Forxiga™ (dapagliflozin)	Bristol Myers Squibb and AstraZeneca	SGLT2 inhibitor	Once-daily tablets for adults with type 2 diabetes
IDegLira (NN9068)	Novo Nordisk	Combination drug therapy	Type 2 diabetes
Invokana (canagliflozin)	Johnson & Johnson	SGLT2 inhibitor	Once-daily tablets for adults with type 2 diabetes
Ipragliflozin L-proline (ASP1941)	Astellas, MSD, and Kotobuki Pharmaceutical	SGLT2 inhibitor	Type 2 diabetes
Luseogliflozin hydrate (TS-071)	Taisho Pharmaceutical	SGLT2 inhibitor	Once-daily for type 2 diabetes
LixiLan (lixisenatide+ insulin glargine)	Sanofi; lixisenatide	Combination drug therapy	Type 2 diabetes
Lyxumia^®^ (lixisenatide)	Sanofi; licensed from Zealand Pharma	GLP-1 agonist	Once-daily for type 2 diabetes
LY2605541 (basal insulin peglispro)	Eli Lilly	Basal insulin analog	Type 1 and 2 diabetes
LY2963016 (new insulin glargine product)	Eli Lilly and Boehringer Ingelheim	Basal insulin	Type 1 and 2 diabetes
Omarigliptin (MK-3102)	Merck & Co.	DPP-4 inhibitor	Once-weekly for adults with type 2 diabetes
Ryzodeg^®^ (insulin degludec + insulin aspart)	Novo Nordisk	Soluble fixed combination of basal insulin with bolus insulin aspart	Once-daily for type 1 and 2 diabetes
Semaglutide (NN9535)	Novo Nordisk	GLP-1 analog	Once-weekly for type 2 diabetes
SYR-472 (trelagliptin succinate)	Takeda Pharmaceuticals and Furiex Pharmaceuticals	DPP-4 inhibitor	Once-weekly oral treatment for type 2 diabetes
Tresiba^®^ (Insulin degludec)	Novo Nordisk	Once-daily basal insulin	Type 1 and 2 diabetes
U300	Sanofi	Insulin glargine	Type 1 and 2 diabetes

**Table 3:** List of new anti-diabetic drugs targeting molecular pathways implicated in diabetes and CVD.

Targets	Mechanism of Action	Leads	Ref.
11β-HSD1 (hydroxysteroid dehydrogenase)	Blocking cortisol	1. INCB13739, MK-0916, 2. BI 135585	[57,58]
G protein-coupled receptor (GPR119)	Increases cAMP signaling	APD5979, MBX-2982	
TGR5 (bile-acid activated GPCR)	cAMP accumulation and enhances GLP-1 secretion (intestine), anti-inflammatory effect (liver)	INT-777	[59]
SIRT1	Improves insulin sensitivity, increases glucose homeostasis, increases mitochondrial capacity	SRT2104, resveratrol	[60]
SGLT-2	Increases kidney-dependent glucose homeostasis	Dapagliflozin, canagliflozin, sergliflozin, remogliflozin, ipragliflozin, and empagliflozin, etc.	[61]
GPR40	Increases incretin secretion, improves glucose tolerance	Modulators	[62]
PPAR-γ	Improves serum lipid profile, glucose homeostasis, insulin sensitivity, reduces inflammation and weight gain	Piolitazone, aleglitazar, glitazones, GFT505	
Tyrosine Kinase	Reduces beta cell apoptosis, enhances insulin secretion, increases beta cell survival, reduces insulin resistance,	Imatinib, Sunitinib, Dasatinib, Sorafenib, Erlotinib	[63]
PPLR	Improves JAK2/STAT5 pathway for glucose uptake	Bromocriptine	
Insulin degrading enzyme (IDE)	Thiol zinc-metalloendopeptidase that cleaves small proteins of diverse sequence	BDM44768, 6bK, NTE-1	[64]
FATP5	Enhances the uptake of long-chain and very long-chain fatty acids into the cells	Chenodiol and Ursodiol	[65]
Sestrin	Enhances hepatic insulin sensitivity	3. Enhances hepatic insulin sensitivity	[66]
Statin	For diabetic dyslipidemia		
Adiponectin	Decreased adiponectin levels are thought to play a central role in the development of type 2 diabetes and obesity	AdipoRon is a novel orally-active small molecule that serves as a potent selective agonist of the AdipoR1 and AdipoR2 adiponectin receptors	[67,68]
Glut4	Triggering the canonical PI3K–AKT pathway is essential and ample to activate exocytosis of GLUT4 storage vesicles to the plasma membrane	Staurosporine is used to promotes GSVs translocation and glucose uptake through the AMPK pathway Rutamarin as a dual inducer of both GLUT4 translocation and expression efficiently ameliorates glucose homeostasis in insulin-resistant mice	[69,70]
PGC-1α	Reduction in PGC-1α triggers insulin resistance and ultimately causes diabetes	Still waiting to synthesize agonist	[71,72]

**Table 4:** Diabetes Mellitus.

Trend Box
Diabetes mellitus is a complex metabolic disruption characterized by chronic hyperglycemia due to deficiencies in insulin production, its systemic release and action, and resistance.
Incident diabetes is also due to changes in performance of multiple key enzymes. Therefore, identifying and understanding key targets at structure-function-dynamics levels and their potential leads and/or drugs, shortcomings, and associated challenges all together can provide a roadmap for the discovery of new selective modulators.
We listed promising therapeutic targets for treatment of T2DM, most of them involving improvements in glucose tolerance.
Novel drugs from various targets have numerous locales of activity and each focus on a few components of the metabolic disorder. These may help pave the way for future type 2 diabetes treatments.
